# Crystal structure of 2,2-di­chloro-1-(piperidin-1-yl)butane-1,3-dione

**DOI:** 10.1107/S2056989014026164

**Published:** 2015-01-01

**Authors:** Markus Schwierz, Helmar Görls, Wolfgang Imhof

**Affiliations:** a University Koblenz-Landau, Institute for Natural Sciences, Universitätsstrasse 1, 56070 Koblenz, Germany; b Friedrich-Schiller-University Jena, Institute of Inorganic and Analytical Chemistry, Humboldtstrasse 8, 07743 Jena, Germany

**Keywords:** crystal structure, 2,2-di­chloro-1-(piperidin-1-yl)butane-1,3-dione, hydrogen bonding, O⋯Cl contacts

## Abstract

In the title compound, C_9_H_13_Cl_2_NO_2_, the piperidine ring shows a chair conformation and the O—C—C—O torsion angle between the carbonyl groups is 183.6 (4)°. In the crystal, mol­ecules are linked into an infinite layer along the *ab* plane by a bifurcated C—H⋯O hydrogen bond between the carbonyl O atom adjacent to the methyl group and one of the methyl­ene groups next to nitro­gen and an additional hydrogen bond of the C—H⋯Cl type. These layers are connected into a three-dimensional supra­molecular arrangement by O⋯Cl contacts [2.8979 (12) and 3.1300 (12) Å].

## Related literature   

For the synthetic procedure, see: Schank (1967 ▸). For a survey concerning weak hydrogen bonds, see: Desiraju & Steiner (1999 ▸). For a description of the nature of inter­molecular inter­actions between chlorine and oxygen, see: Lommerse *et al.* (1996 ▸). For the X-ray structure of the starting compound, see: Schwierz *et al.* (2014 ▸).
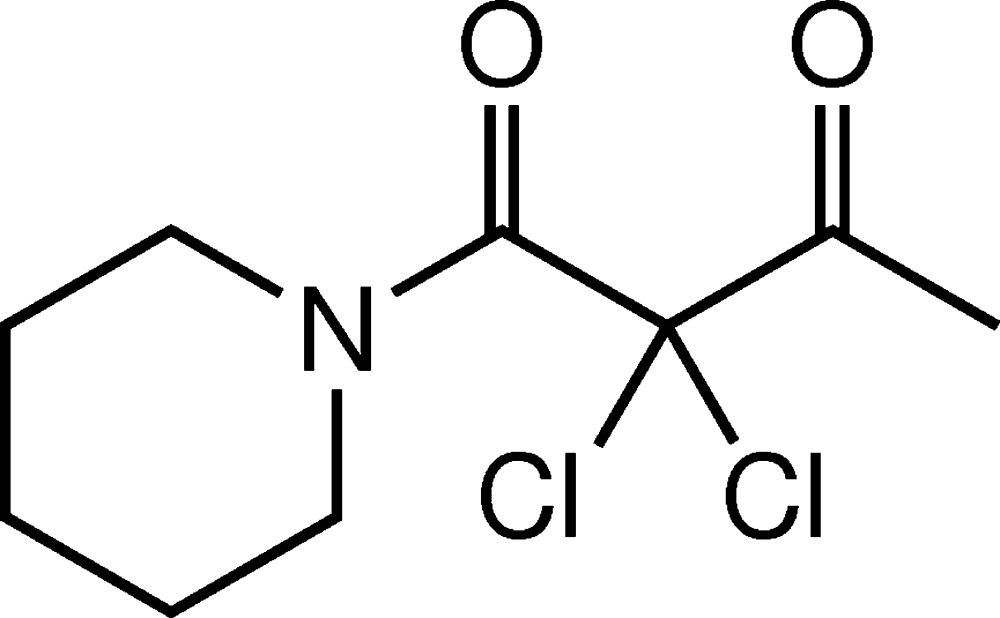


## Experimental   

### Crystal data  

C_9_H_13_Cl_2_NO_2_
*M* *_r_* = 238.10
Monoclinic, 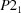

*a* = 5.9548 (3) Å
*b* = 10.5510 (4) Å
*c* = 8.5747 (3) Å
β = 100.568 (2)°
*V* = 529.60 (4) Å^3^
*Z* = 2
Mo *K*α radiation
μ = 0.59 mm^−1^
*T* = 133 K
0.07 × 0.05 × 0.02 mm

### Data collection  

Nonius KappaCCD diffractometer
Absorption correction: multi-scan (*SADABS*; Bruker, 2002 ▸) *T*_min_ = 0.616, *T*_max_ = 0.746
3076 measured reflections
2402 independent reflections
2085 reflections with *I* > 2σ(*I*)
*R*_int_ = 0.030

### Refinement  

*R*[*F* ^2^ > 2σ(*F* ^2^)] = 0.024
*wR*(*F* ^2^) = 0.059
*S* = 1.09
2402 reflections
128 parameters
1 restraint
All H-atom parameters refined
Δρ_max_ = 0.33 e Å^−3^
Δρ_min_ = −0.21 e Å^−3^
Absolute structure: Flack (1983 ▸), 1115 Friedel pairs
Absolute structure parameter: 0.08 (4)

### 

Data collection: *COLLECT* (Nonius, 1998 ▸); cell refinement: *DENZO* (Otwinowski & Minor, 1997 ▸); data reduction: *DENZO*; program(s) used to solve structure: *SHELXS97* (Sheldrick, 2008 ▸); program(s) used to refine structure: *SHELXL97* (Sheldrick, 2008 ▸); molecular graphics: *ORTEP-3 for Windows* (Farrugia, 2012 ▸) and *Mercury* (Macrae *et al.*, 2006 ▸); software used to prepare material for publication: *SHELXL97*.

## Supplementary Material

Crystal structure: contains datablock(s) I, New_Global_Publ_Block. DOI: 10.1107/S2056989014026164/hg5420sup1.cif

Structure factors: contains datablock(s) I. DOI: 10.1107/S2056989014026164/hg5420Isup2.hkl

Supporting information file. DOI: 10.1107/S2056989014026164/hg5420Isup3.cml

**Fig. 1.**
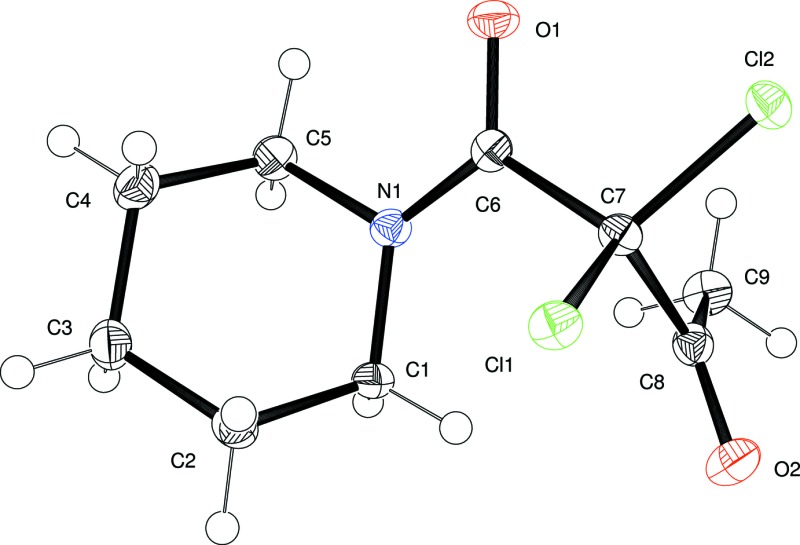
: Molecular structure of the title compound with thermal ellipsoids drawn at the 50% probability level.

. DOI: 10.1107/S2056989014026164/hg5420fig1.tif
Mol­ecular structure of the title compound with thermal ellipsoids drawn at the 50% probability level.

**Fig. 2.**
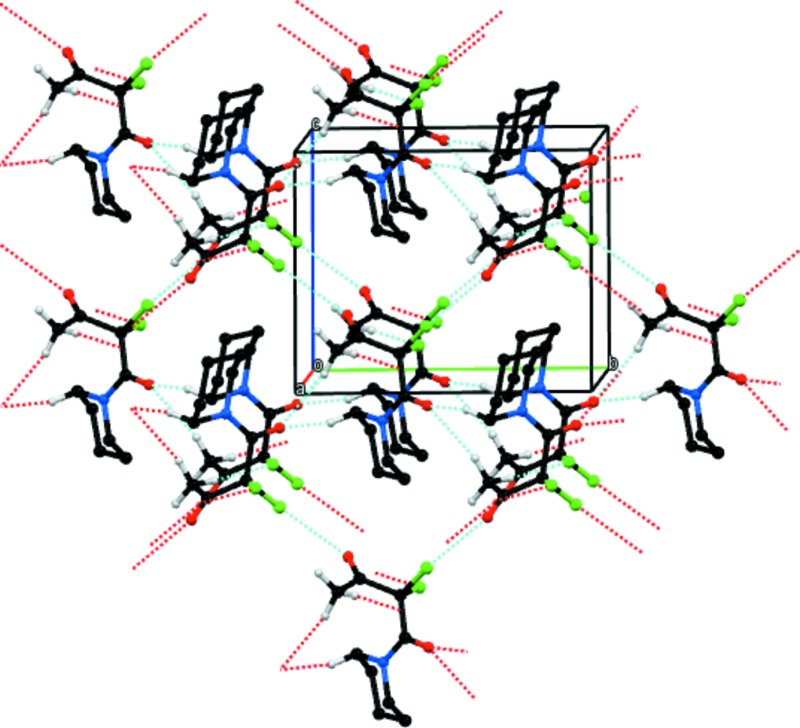
: Crystal structure of the title compound showing a 3D supramolecular network built up by C–H···O and C–H···Cl hydrogen bonds and chlorine oxygen contacts. Hydrogen atoms at piperidine residues that are not involved in hydrogen bonding are omitted for the sake of clarity.

. DOI: 10.1107/S2056989014026164/hg5420fig2.tif
Crystal structure of the title compound showing a 3D supra­molecular network built up by C–H⋯O and C–H⋯Cl hydrogen bonds and chlorine oxygen contacts. Hydrogen atoms at piperidine residues that are not involved in hydrogen bonding are omitted for the sake of clarity.

CCDC reference: 1036594

Additional supporting information:  crystallographic information; 3D view; checkCIF report

## Figures and Tables

**Table 1:** Hydrogen-bond geometry (, )

*D*H*A*	*D*H	H*A*	*D* *A*	*D*H*A*
C1H1*A*O1^i^	0.99	2.56	3.413(2)	145
C9H9*C*O1^i^	0.98	2.53	3.494(2)	168
C9H9*C*Cl1^ii^	0.98	2.79	3.770(2)	176

Symmetry codes: (i) 
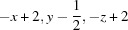
; (ii) 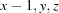.

## supplementary crystallographic information

### S1. Comment

The title compound is an intermediate in the synthesis of 2,2-dimethoxy-1-(pyridin-2-yl)ethanone and has been synthesized from 1-(piperidin-1-yl)butane-1,3-dione (Schwierz *et al.*, 2014) following a modified procedure (Schank, 1967). As it is expected the piperidine ring shows a chair conformation and the amide substructure is planar (Figure 1). The dihedral angle O1—C6—C8—O2 between the carbonyl groups measures to 183.6 (4)°. The C—O bond of the amide carbonyl group is slightly elongated with respect to the other carbonyl group due to delocalization of the nitrogen lone pair (C6—O1 1.221 (3) Å *versus*. C8—O2 1.205 (3) Å). In the crystal structure, molecules are linked to infinite layers along the *ab* plane by a bifurcated hydrogen bond between one of the carbonyl oxygen atoms (O1) towards the methyl group and one of the methylene groups next to nitrogen and an additional hydrogen bond of the C—H···Cl type (Desiraju & Steiner, 1999). In addition, these layers are connected to a 3D supramolecular arrangement by oxygen chlorine contacts (Lommerse *et al.*, 1996).

### S2. Experimental

25.4 g (0.15 mol) 1-(piperidin-1-yl)butane-1,3-dione were dissolved in 70 ml dichloromethane. To this solution, 24.3 ml (40.6 g, 0.3 mol) sulfuryl dichloride was added dropwise and the resulting mixture refluxed for 5 h. After cooling to room temperature 30 ml diethylether were added and the solution washed with brine (3 ×20 ml), dried over CaCl_2_, filtered and evaporated to dryness. The resulting highly viscous product was distilled *in vacuo* (0.2 mbar). Condensation of the distillate into a Schlenk tube cooled with liquid nitrogen yielded crystalline material suitable for X-ray diffraction (Combined yield of all fractions: 32.5 g, 91%).

### S3. Refinement

Hydrogen atoms have been calculated into idealized positions with C–H = 0.98 - 0.99 Å . Methylene and methyl hydrogen atoms were refined with *U*_iso_ = 1.2 *U*_eq_(C) and 1.5 *U*_eq_(C) respectively.

### Crystal data

**Table:**

C_9_H_13_Cl_2_NO_2_	*Z* = 2
*M**_r_* = 238.10	*F*(000) = 248
Monoclinic, *P*2_1_	*D*_x_ = 1.493 Mg m^−^^3^
Hall symbol: P 2yb	Mo *K*α radiation, λ = 0.71073 Å
*a* = 5.9548 (3) Å	µ = 0.59 mm^−^^1^
*b* = 10.5510 (4) Å	*T* = 133 K
*c* = 8.5747 (3) Å	Prism, colourless
β = 100.568 (2)°	0.07 × 0.05 × 0.02 mm
*V* = 529.60 (4) Å^3^	

### Data collection

**Table:**

Nonius KappaCCD diffractometer	2402 independent reflections
Radiation source: fine-focus sealed tube	2085 reflections with *I* > 2σ(*I*)
Graphite monochromator	*R*_int_ = 0.030
phi– + ω–scan	θ_max_ = 27.5°, θ_min_ = 2.4°
Absorption correction: multi-scan (*SADABS*; Bruker, 2002)	*h* = −5→7
*T*_min_ = 0.616, *T*_max_ = 0.746	*k* = −13→13
3076 measured reflections	*l* = −11→11

### Refinement

**Table:**

Refinement on *F*^2^	Secondary atom site location: difference Fourier map
Least-squares matrix: full	Hydrogen site location: mixed
*R*[*F*^2^ > 2σ(*F*^2^)] = 0.024	All H-atom parameters refined
*wR*(*F*^2^) = 0.059	*w* = 1/[σ^2^(*F*_o_^2^) + (0.0215*P*)^2^ + 0.1164*P*] where *P* = (*F*_o_^2^ + 2*F*_c_^2^)/3
*S* = 1.09	(Δ/σ)_max_ = 0.001
2402 reflections	Δρ_max_ = 0.33 e Å^−^^3^
128 parameters	Δρ_min_ = −0.21 e Å^−^^3^
1 restraint	Absolute structure: Flack (1983), 1115 Friedel pairs
Primary atom site location: structure-invariant direct methods	Absolute structure parameter: 0.08 (4)

### Special details

**Table:**

Geometry. All e.s.d.'s (except the e.s.d. in the dihedral angle between two l.s. planes) are estimated using the full covariance matrix. The cell e.s.d.'s are taken into account individually in the estimation of e.s.d.'s in distances, angles and torsion angles; correlations between e.s.d.'s in cell parameters are only used when they are defined by crystal symmetry. An approximate (isotropic) treatment of cell e.s.d.'s is used for estimating e.s.d.'s involving l.s. planes.
Refinement. Refinement of *F*^2^ against ALL reflections. The weighted *R*-factor *wR* and goodness of fit *S* are based on *F*^2^, conventional *R*-factors *R* are based on *F*, with *F* set to zero for negative *F*^2^. The threshold expression of *F*^2^ > σ(*F*^2^) is used only for calculating *R*-factors(gt) *etc*. and is not relevant to the choice of reflections for refinement. *R*-factors based on *F*^2^ are statistically about twice as large as those based on *F*, and *R*- factors based on ALL data will be even larger.

### Fractional atomic coordinates and isotropic or equivalent isotropic displacement parameters (Å^2^)

**Table:**

	*x*	*y*	*z*	*U*_iso_*/*U*_eq_	
Cl1	1.44673 (6)	0.94886 (3)	0.75108 (4)	0.01711 (9)	
Cl2	0.97291 (6)	1.00243 (3)	0.62597 (4)	0.01824 (9)	
O1	0.9936 (2)	1.00543 (12)	0.95581 (13)	0.0203 (2)	
O2	1.2365 (2)	0.72093 (11)	0.60201 (14)	0.0228 (3)	
N1	1.2241 (2)	0.83853 (12)	1.03862 (15)	0.0149 (3)	
C1	1.3935 (3)	0.73987 (14)	1.02007 (18)	0.0156 (3)	
H1A	1.4037	0.7314	0.9066	0.019*	
H1B	1.3440	0.6574	1.0572	0.019*	
C2	1.6274 (3)	0.77486 (15)	1.11579 (19)	0.0183 (3)	
H2A	1.6857	0.8511	1.0689	0.022*	
H2B	1.7358	0.7046	1.1101	0.022*	
C3	1.6153 (3)	0.80114 (17)	1.28938 (19)	0.0211 (3)	
H3A	1.5787	0.7217	1.3409	0.025*	
H3B	1.7656	0.8316	1.3458	0.025*	
C4	1.4336 (3)	0.90029 (16)	1.30135 (19)	0.0208 (3)	
H4A	1.4784	0.9824	1.2603	0.025*	
H4B	1.4203	0.9120	1.4139	0.025*	
C5	1.2036 (3)	0.85828 (15)	1.20554 (18)	0.0183 (3)	
H5A	1.1546	0.7785	1.2501	0.022*	
H5B	1.0867	0.9238	1.2120	0.022*	
C6	1.1183 (3)	0.91946 (13)	0.92819 (18)	0.0140 (3)	
C7	1.1618 (3)	0.90173 (14)	0.75549 (18)	0.0145 (3)	
C8	1.1108 (3)	0.76732 (14)	0.68101 (18)	0.0160 (3)	
C9	0.8979 (3)	0.70565 (15)	0.7144 (2)	0.0202 (3)	
H9D	0.8409	0.6448	0.6300	0.030*	
H9C	0.7814	0.7705	0.7187	0.030*	
H9B	0.9321	0.6613	0.8164	0.030*	

### Atomic displacement parameters (Å^2^)

**Table:**

	*U*^11^	*U*^22^	*U*^33^	*U*^12^	*U*^13^	*U*^23^
Cl1	0.01400 (17)	0.01793 (17)	0.02027 (18)	−0.00249 (13)	0.00546 (13)	−0.00093 (14)
Cl2	0.01831 (18)	0.01753 (16)	0.01794 (17)	0.00204 (14)	0.00086 (13)	0.00373 (14)
O1	0.0207 (6)	0.0196 (5)	0.0218 (5)	0.0062 (5)	0.0069 (5)	−0.0008 (5)
O2	0.0234 (6)	0.0232 (6)	0.0229 (6)	−0.0011 (5)	0.0071 (5)	−0.0076 (5)
N1	0.0130 (6)	0.0157 (6)	0.0162 (6)	0.0009 (5)	0.0030 (5)	0.0001 (5)
C1	0.0147 (7)	0.0140 (7)	0.0177 (8)	0.0028 (6)	0.0020 (6)	−0.0007 (5)
C2	0.0133 (7)	0.0199 (7)	0.0212 (8)	0.0014 (6)	0.0019 (6)	0.0002 (6)
C3	0.0184 (8)	0.0259 (8)	0.0178 (8)	−0.0008 (6)	−0.0001 (6)	0.0001 (6)
C4	0.0226 (9)	0.0232 (8)	0.0171 (7)	−0.0012 (7)	0.0050 (6)	−0.0035 (6)
C5	0.0179 (8)	0.0221 (8)	0.0160 (7)	0.0006 (6)	0.0062 (6)	0.0011 (6)
C6	0.0101 (7)	0.0151 (7)	0.0169 (7)	−0.0040 (5)	0.0026 (6)	−0.0014 (5)
C7	0.0115 (7)	0.0153 (7)	0.0172 (7)	−0.0009 (6)	0.0041 (6)	0.0009 (6)
C8	0.0169 (8)	0.0148 (7)	0.0152 (7)	0.0004 (6)	0.0001 (6)	0.0008 (6)
C9	0.0187 (8)	0.0177 (7)	0.0246 (8)	−0.0040 (7)	0.0047 (7)	−0.0016 (6)

### Geometric parameters (Å, º)

**Table:**

Cl1—C7	1.7752 (16)	C3—H3A	0.9900
Cl2—C7	1.7802 (15)	C3—H3B	0.9900
O1—C6	1.2226 (19)	C4—C5	1.528 (2)
O2—C8	1.202 (2)	C4—H4A	0.9900
N1—C6	1.3431 (19)	C4—H4B	0.9900
N1—C5	1.4734 (19)	C5—H5A	0.9900
N1—C1	1.4782 (19)	C5—H5B	0.9900
C1—C2	1.527 (2)	C6—C7	1.561 (2)
C1—H1A	0.9900	C7—C8	1.562 (2)
C1—H1B	0.9900	C8—C9	1.499 (2)
C2—C3	1.529 (2)	C9—H9D	0.9800
C2—H2A	0.9900	C9—H9C	0.9800
C2—H2B	0.9900	C9—H9B	0.9800
C3—C4	1.522 (2)		
			
C6—N1—C5	118.90 (12)	H4A—C4—H4B	108.2
C6—N1—C1	127.81 (13)	N1—C5—C4	109.73 (13)
C5—N1—C1	112.57 (12)	N1—C5—H5A	109.7
N1—C1—C2	110.19 (12)	C4—C5—H5A	109.7
N1—C1—H1A	109.6	N1—C5—H5B	109.7
C2—C1—H1A	109.6	C4—C5—H5B	109.7
N1—C1—H1B	109.6	H5A—C5—H5B	108.2
C2—C1—H1B	109.6	O1—C6—N1	123.93 (14)
H1A—C1—H1B	108.1	O1—C6—C7	119.03 (13)
C1—C2—C3	111.45 (13)	N1—C6—C7	117.04 (12)
C1—C2—H2A	109.3	C6—C7—C8	116.30 (12)
C3—C2—H2A	109.3	C6—C7—Cl1	108.20 (10)
C1—C2—H2B	109.3	C8—C7—Cl1	111.13 (11)
C3—C2—H2B	109.3	C6—C7—Cl2	108.94 (10)
H2A—C2—H2B	108.0	C8—C7—Cl2	103.51 (10)
C4—C3—C2	110.58 (13)	Cl1—C7—Cl2	108.45 (8)
C4—C3—H3A	109.5	O2—C8—C9	124.54 (14)
C2—C3—H3A	109.5	O2—C8—C7	120.38 (14)
C4—C3—H3B	109.5	C9—C8—C7	115.07 (13)
C2—C3—H3B	109.5	C8—C9—H9D	109.5
H3A—C3—H3B	108.1	C8—C9—H9C	109.5
C3—C4—C5	110.03 (14)	H9D—C9—H9C	109.5
C3—C4—H4A	109.7	C8—C9—H9B	109.5
C5—C4—H4A	109.7	H9D—C9—H9B	109.5
C3—C4—H4B	109.7	H9C—C9—H9B	109.5
C5—C4—H4B	109.7		

### Hydrogen-bond geometry (Å, º)

**Table:**

*D*—H···*A*	*D*—H	H···*A*	*D*···*A*	*D*—H···*A*
C1—H1*A*···O1^i^	0.99	2.56	3.413 (2)	145
C9—H9*C*···O1^i^	0.98	2.53	3.494 (2)	168
C9—H9*C*···Cl1^ii^	0.98	2.79	3.770 (2)	176

Symmetry codes: (i) −*x*+2, *y*−1/2, −*z*+2; (ii) *x*−1, *y*, *z*.
