# Supplementary figures and images for: Crystal structure of 2,2-di­chloro-1-(piperidin-1-yl)butane-1,3-dione
Source: Acta Crystallogr E Crystallogr Commun. 2015 Jan 1;71(Pt 1):o19. doi: 10.1107/S2056989014026164 (PMC4331874; doi:10.1107/S2056989014026164)

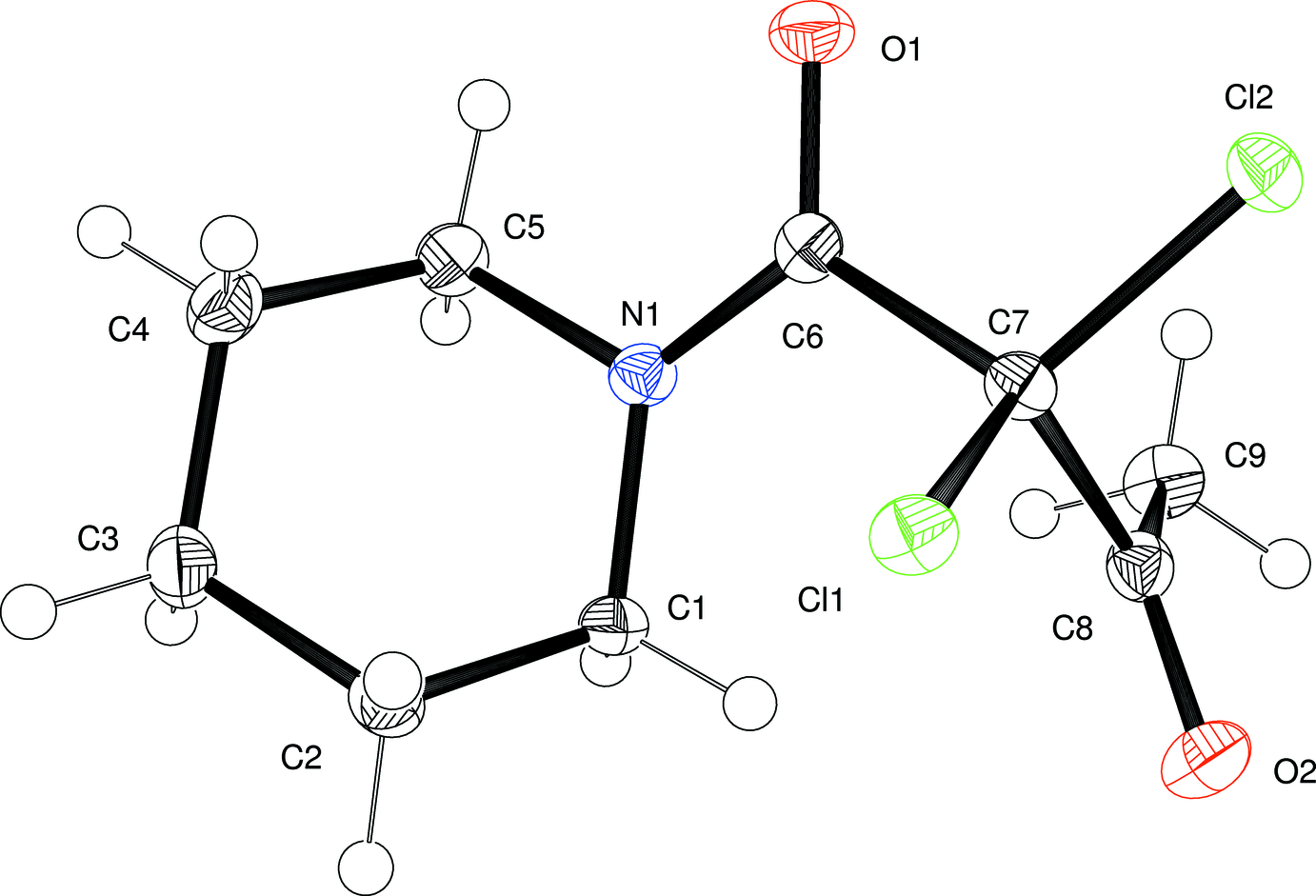

Supplement: Supplementary file 4 [file e-71-00o19-fig1.tif]

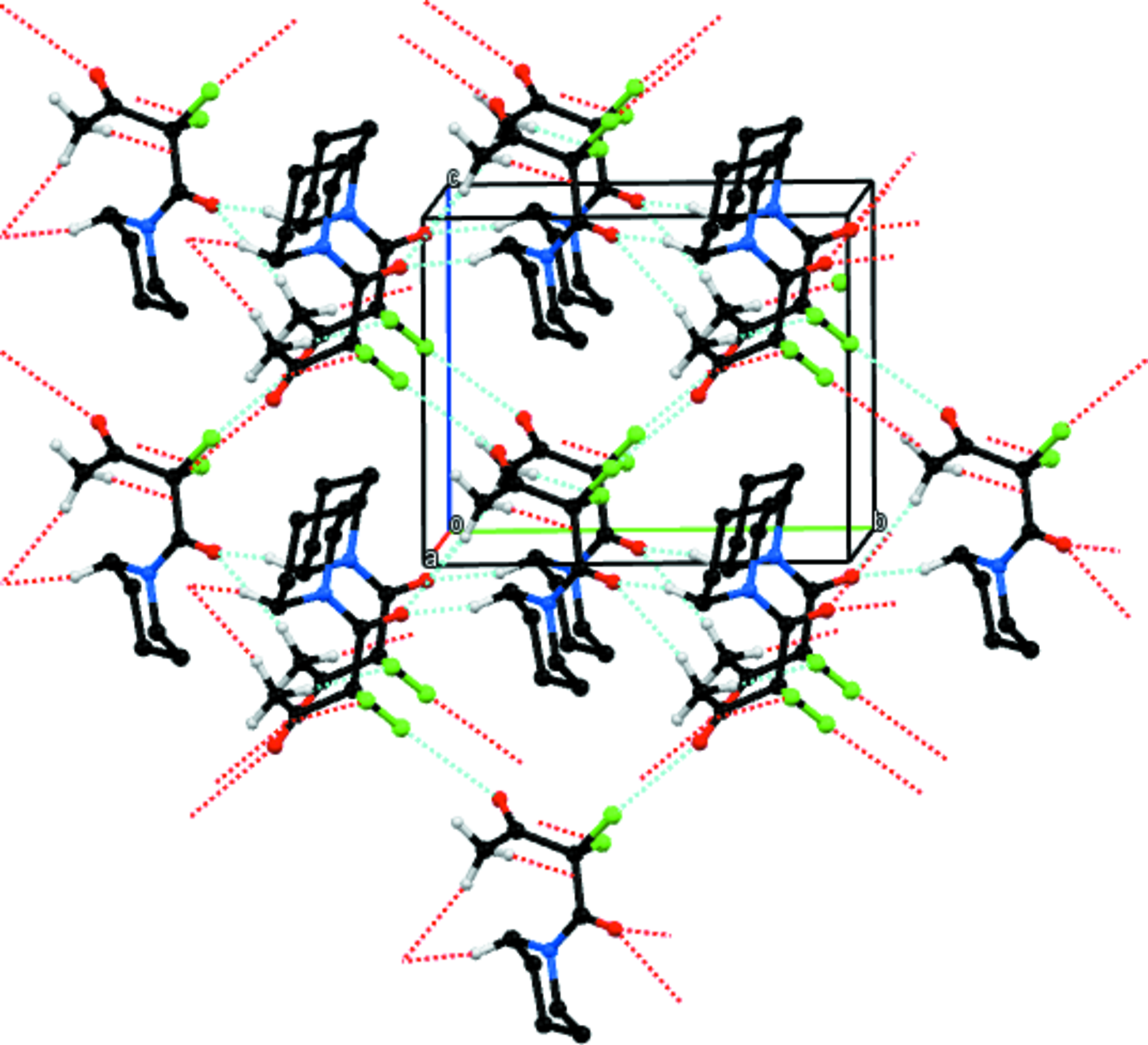

Supplement: Supplementary file 5 [file e-71-00o19-fig2.tif]
